# Online Simulation Training of Child Sexual Abuse Interviews With Feedback Improves Interview Quality in Japanese University Students

**DOI:** 10.3389/fpsyg.2020.00998

**Published:** 2020-05-26

**Authors:** Shumpei Haginoya, Shota Yamamoto, Francesco Pompedda, Makiko Naka, Jan Antfolk, Pekka Santtila

**Affiliations:** ^1^Faculty of Arts and Sciences, New York University (NYU) Shanghai, Shanghai, China; ^2^Life Skill Education Institute, Graduate School of Hosei University, Tokyo, Japan; ^3^Faculty of Arts, Psychology, and Theology, Åbo Akademi University, Turku, Finland; ^4^School of Natural & Social Sciences, University of Gloucestershire, Cheltenham, United Kingdom; ^5^Department of Comprehensive Psychology, Ritsumeikan University, Kyoto, Japan; ^6^Turku Brain and Mind Center, Turku, Finland

**Keywords:** child sexual abuse (CSA), internet, investigative interviewing, simulation training, remote learning, serious gaming, virtual reality

## Abstract

Although previous research has confirmed the effectiveness of simulated child sexual abuse interviews with feedback, its validation is limited to Western contexts and face-to-face settings. The present study aims to extend this research to non-Western and online/remote training conditions. Thirty-two Japanese undergraduate students were randomly assigned to a control or feedback group. The feedback group conducted a set of six online simulated child sexual abuse interviews while receiving feedback after each interview in an attempt to improve the quality of their questioning style. The feedback consisted of the outcome of the alleged cases and the quality of the questions asked in the interviews. The control group conducted the interviews without feedback. The feedback (vs. control) increased the proportion of recommended questions (first interview: 45%; last interview: 65% vs. first: 43%; last: 42%, respectively) by using fewer not-recommended questions and eliciting fewer incorrect details. Furthermore, only participants in the feedback group (7 out of 17) demonstrated a reliable change in the proportion of recommended questions. The present study explores the efficacy of simulated interview training with avatars in a different cultural setting and in the context of remote administration. The differences between the present study and previous research are discussed in light of cultural and logistical aspects.

## Introduction

In most cases of child sexual abuse (CSA), victims tend not to disclose the abuse immediately and clear evidence is rarely found (World Health Organization, 2003). In fact, physical evidence of CSA is available in only 10–35% of alleged cases (DeJong, 1992; Dubowitz et al., 1992; Elliott and Briere, 1994; DiPietro et al., 1997), and the child’s testimony is often the only evidence in the investigation (Lamb, 1995; Leander, 2010). Given the importance of interviews with alleged victims, it is unfortunate that children are susceptible to suggestion (Ceci and Bruck, 1993), and their testimony may be distorted by leading interviewing methods using option-posing, forced choice, suggestive, and repeated questions (Roberts and Lamb, 1999; Finnilä et al., 2003; Volpini et al., 2016). Meanwhile, extensive research has confirmed that children are more likely to provide trustworthy information in response to open-ended questions (e.g., Allwood et al., 2008). Thus, in forensic settings, the use of open-ended questions is imperative.

Aiming at guiding the child interview in a manner which elicits undistorted information from children, the National Institute of Child Health and Human Development (NICHD) protocol was introduced (Hershkowitz et al., 2007). It is a structured interview protocol that covers all the steps of an investigative interview, including introduction, rapport-building, and free-recall. The use of this protocol results in interviewers using more open-ended questions and eliciting more disclosures from children (Orbach et al., 2000; Sternberg et al., 2001). In training interviewers to abide by the NICHD protocol, it is considered important to provide continuous support (Lamb et al., 2007), and recent research has emphasized the importance of providing feedback (Price and Roberts, 2011; Rischke et al., 2011). Most previous research has provided interviewer feedback on interviews with real children. However, this kind of feedback is time-consuming and costly, and it may be difficult to maintain it outside of research projects (Pompedda et al., 2015). In addition, research based on real interviews has only reflected improvement in the use of appropriate question types. In contrast, it is almost impossible to know whether the interviewers get better at finding out the truth, as what actually happened in the alleged case is not known. For example, it may be that the interviewer correctly determines that the child has been sexually abused. However, some of the details regarding what exactly had happened could still be wrong, and there might be no way of determining this later.

To overcome the issues stated above, Pompedda et al. (2015) proposed using simulated training in CSA interviews using avatars (hereafter referred to as Avatar Training). Avatar Training is a training protocol designed to teach the use of open questions in realistic interview exercises with avatars. In these simulated interviews, the interviewer can elicit information from the avatar by asking questions freely and orally as if they were interviewing an actual child. Currently, in many training programs, roleplay between adult trainees is used as an exercise (e.g., Powell and Wright, 2008; Powell et al., 2011; Naka, 2015). However, this method has potential limitations in terms of realism. For instance, adults who play the role of the interviewee are often not good at imitating the age-specific cognitive abilities of children (Powell et al., 2011). In contrast, Avatar Training is a training method that provides the opportunity to practice CSA interviews using avatars that behave based on how children have responded to the same type of questioning in previous research on children’s memory and suggestibility. Media equation theory (Szczuka et al., 2019) suggests that people interact socially and naturally with avatars; while they are aware that the avatars are not real human beings, they nevertheless behave as if they were.

In the first study on Avatar Training, Pompedda et al. (2015) showed that simulated interviews followed by a report on both the outcomes (a description of what actually happened in the case) and the question types (explanations about two recommended questions and two not-recommended questions asked by the interviewer) resulted in more open-ended questions and less option-posing and suggestive questions asked by interviewers than interviews without feedback. Since the first study examined only the simultaneous use of outcome and question type feedback, Pompedda et al. (2017) compared the following four conditions to verify the independent effects of the two types of feedback: (1) providing outcome and question type feedback, (2) providing outcome feedback, (3) providing question feedback, and (4) providing no feedback. The results showed that the combination of outcome and question type feedback had the strongest effect on increasing the proportion of open-ended questions and making correct conclusions in the alleged cases. Further, Krause et al. (2017) examined the effect of reflection on the questions asked in the interview as a new intervention and found no difference between the condition providing reflection together with feedback and the group given feedback only. Summarizing the findings obtained so far, the combination of feedback on both the outcome and question types within the Avatar Training seems effective in improving the interview quality (Pompedda, 2018).

In terms of possible cultural differences, Avatar Training has a potential constraint in its applicability since previous studies have examined its effectiveness exclusively in Western contexts such as Finland (Krause et al., 2017) and Italy (Pompedda et al., 2015, 2017). The use of more open-ended questions to elicit more disclosures from children is equally important in the Asian context (Naka, 2001, 2012; Yi et al., 2015). In addition, these countries have utilized the NICHD protocol that guides interviewers to use the same basic questioning skills that are also acquired by Avatar Training (La Rooy et al., 2015). For example, training courses based on the NICHD protocol have been implemented in Japan since 2007, and more than 2,500 people had participated in them up until 2014 (Naka, 2015). In the Avatar Training, the Japanese Avatar scenarios are slightly adjusted to take into consideration cultural and societal differences between Japanese and Western contexts. In addition, there are potential differences in learning styles that might affect the efficacy of the current setup (Oxford and Anderson, 1995). Therefore, the present study aims to extend the validity of Avatar Training to different cultural groups by conducting simulated interviews with feedback in Japan.

So far, in terms of administering interview training, most training sessions have been face-to-face. However, face-to-face training poses problems that may undermine the availability and effectiveness of training. For instance, the trainer and trainees need to travel and be away from work to get together at the same place to conduct the training. In addition, face-to-face training entails many logistics since it is often administered to a large group of trainees. Moreover, in a large group, trainees have less opportunity to practice and receive feedback from the trainer. In contrast, remote training using audio or video calls may sidestep the logistical problems of face-to-face training without limiting the location of the trainer and trainee. Even with regards to training efficacy, recent research has illustrated that training might not necessarily have to be conducted face-to-face. For example, studies comparing face-to-face and audio interactions showed no difference with regards to psychotherapy (Day and Schneider, 2002) and collaborative learning (Roseth et al., 2011). Even for trainees to learn to use open questions, text-based feedback provided during online training improved interviewers’ questioning skills (Powell et al., 2016). In the Avatar Training, the desktop application that offers the simulated interview used in previous research has been redeveloped as a web application that runs on a web browser and can be used remotely for conducting training by combining it with voice call. Accordingly, in addition to the cross-cultural verification mentioned above, the present study aimed to validate the effectiveness of the Avatar Training performed remotely.

The present study tested the following four hypotheses:

1. Avatar Training with feedback (vs. without feedback) will improve interview quality (i.e., the number of recommended and not recommended questions, and the proportion of recommended questions).
2. Improved interview quality in the Avatar Training with feedback (vs. without feedback) will result in an increase in correct details elicited from avatars and a decrease in incorrect details.
3. The difference between the feedback and the no feedback groups will become statistically significant from the fourth interview onward in indicators of interview quality and elicited details.
4. The proportion of correct conclusions made by interviewers will be improved in the Avatar Training with feedback (vs. without feedback).

## Materials and Methods

### Participants

The participants were 32 (23 women) university undergraduates (*M*_age_ = 20.5, *SD* = 0.6) who completed the experiment for either course credit or 1,000 JPY. They were randomly allocated into either the feedback (*n* = 17) or the control group (*n* = 15). None had experience of CSA investigations, and three had parenting experience. The board of research ethics at the Department of Psychology, Hosei University (Japan) approved the studies before the data collection commenced. All subjects provided written informed consent in accordance with the Declaration of Helsinki.

### Design

The present study employed a 2 (feedback and no-feedback: between-participants) × 6 (number of interviews from 1 to 6: within-participants) mixed design. Participants were randomly assigned to either feedback or no-feedback conditions.

### Materials

#### Avatar Training

**FIGURE 1:**
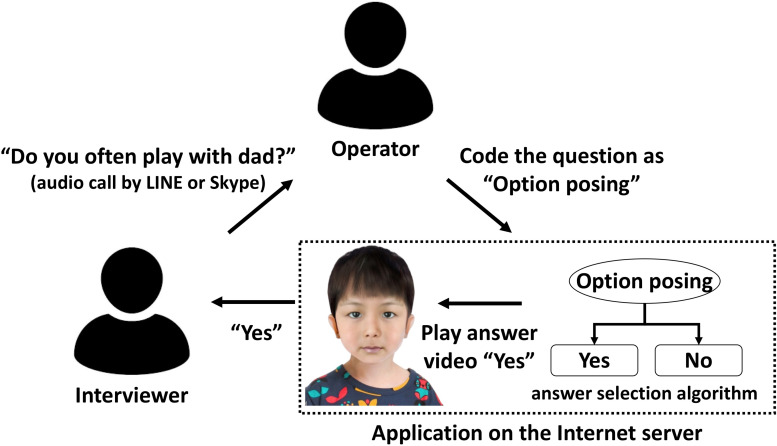
Example of the structure of the Avatar Training. The avatar image displayed is not an exact image of anyone since the designer created the images of avatars by combining elements (e.g., eyes, nose, mouth, ears, hairstyle, facial outline) from two or more different individuals.

The simulated interview application used for the experiment, Avatar Training, included a total of 16 avatars consisting of two avatars in every one of the eight conditions that are different in age (4 and 6 years), gender (women and men), and the presence or absence of abuse. The Avatar Training had an answer selection algorithm based on the empirical literature (see Pompedda et al., 2015 for details) of the relationship between question types (e.g., invitations, facilitators) and the contents of the child avatar’s answer. During training, the operator coded the questions asked by the interviewer to one of the question types, and the answer selection algorithm process then automatically selected and provided the avatar’s responses contained in a video clip (see Figure 1).

In the Japanese version of Avatar Training, in consideration of cultural differences, we made modifications to the background narratives of the alleged abuse cases that were presented to the interviewer before the training, the avatar’s answers to the interviewer, and the outcome presented after the training (i.e., a description of what actually happened in the case). For instance, the environment in which a particular abuse allegation arose was changed from a church to a local children’s group. We intended to minimize the changes in the Japanese version from the contents used in Finland and Italy to maintain consistency between them. After modifications, police investigators who were in charge of CSA investigations in the Japanese Police evaluated the realism of the scenarios in relation to actual CSA cases in Japan. Further modifications were made in line with their advice.

#### Question Type Coding

Question type coding (see Table 1) utilized the classification of questions presented in previous research (Sternberg et al., 1996; Korkman et al., 2006; Pompedda et al., 2015). The coding of questions was carried out by one of the three operators. To ensure that the operators used the same coding rules, the three pairs of operators repeatedly conducted simulated interviews while exchanging the roles of the interviewer and operator. To calculate the inter-rater reliability of the coding, an additional trained undergraduate student who was blinded to the condition of participants coded 25% (48 interviews by 8 participants, 1,475 questions) of completed interviews. The total percentage of agreement in coding between the student and the operators was 74%, 95% *CI* (70%, 78%), with a Cohen’s kappa (κ: Cohen, 1988) of 0.634, *p* < 0.001, 95% *CI* [0.595, 0.673]. This can be considered substantial agreement (Landis and Koch, 1977) and similar to rates of agreement obtained in previous research (Krause et al., 2017).

**TABLE 1:** Description and examples by question types.

Question types	Description	Examples

Recommended questions		
Invitations	Open-ended and non-suggestive questions that elicit free narrative from children.	“Tell me everything that happened from the beginning to the end” “Tell me about your family”
Facilitators	Non-suggestive questions that promote further narrative about the content previously mentioned.	“What happened after that?” “Go ahead” “Ok”
Directive	Questions that focus the children’s attention on the content the child has already mentioned for further explanation.	“Where did you go with your mom?” “What game did you play?”

**Not recommended questions**		

Option posing	Closed questions that focus the children’s attention on content that the child had not yet mentioned without implying a specific type of answer.	“Do you play with dad?”
Specific suggestive	Questions that indicate what kind of answer is expected by assuming details that children have not mentioned.	“Did your dad do something bad to you?” “Is your dad a bad person?”
Unspecific suggestive	Questions that indicate what kind of answer is expected without assuming details that children have not mentioned.	“I know that I have something to talk about, tell me!”
Repetition	Questions continuously asking what the interviewer has already asked.	
Too long	Questions that ask for more than one detail at once.	“Where were you with your father, what were you doing after that?”
Unclear	Questions that contain words that are too difficult for the children’s cognitive level or that are grammatically unclear.	“What is the relationship between mom and dad?”
Multiple choice	Questions that focus the children’s attention on specific answers, or force them to choose among options.	“Did you go practicing football with Kanta or Miura?”
Time	Questions that rely on temporal cognitive processes that are underdeveloped in children under 6 years of age.	“When did your mom leave the park?”
Feelings	Questions that rely on emotional cognitive processes underdeveloped in children under 6 years of age.	“What do you think of your Grandpa?”
Fantasy	Questions that encourage children’s fantasies and may produce inaccurate answers.	“If you were your dad, what did you do?”

#### Answer Selection

Avatar Training is equipped with an algorithm that has answers with predefined probabilities for being given as a response to the different question types. The exact algorithms were different for the 4 and 6-year-old avatars based again on empirical findings on child memory and suggestibility in interviews (see Pompedda et al., 2015 for details).

Information related to the alleged case (hereafter referred to as relevant details) and information unrelated to the case (hereafter referred to as neutral details) was provided only in response to recommended questions. Each avatar had nine relevant details and nine neutral details contained in their “memory,” and the algorithm provided one of them as a response to each recommended question according to a predefined probability. The probability that relevant and neutral details were provided when the interviewer asked a recommended question was set as 12.5% at age 4 and 25% at age 6, respectively. Relevant and neutral details were provided in a fixed order, and the last four of the relevant details included the information needed to reach the correct conclusions regarding the case. To enhance the realism of the interview, each avatar had a series of side details organized by keywords. The information was displayed to the operator as a list during the interview, and the operator manually played the answer from the list corresponding to the interviewer’s question. For instance, if the interviewer asked, “What do you always do with your grandfather?,” the operator would select the answer at the top of the list of side details under the keyword “grandfather” instead of launching the “recommended question” algorithm. Side details were provided only for recommended questions, and answers corresponding to a particular keyword were provided to the interviewer until all the listed videos have been provided. Meanwhile, the algorithm for recommended questions was launched for questions that do not correspond to any of the keywords (or questions with a keyword for which all the corresponding answers had already been provided to the interviewer) and questions about the alleged case.

Incorrect details (inconsistent information with details that avatars hold) could be generated if the interviewer asked not- recommended questions. Information held by avatars was predefined as a set of answers, and incorrect details were detected as inconsistencies with that. For example, an incorrect detail could be created should the interviewer ask, “Did dad hurt you?” to an avatar that has not been abused and the answer selection algorithm return a “yes” with a probability of 30% (4-year-old avatars).

#### Procedure

Each experimental session took 2–2.5 h. The experiment was conducted remotely using LINE, a social networking service provided by LINE Corporation, or Skype, an Internet telephone service provided by Microsoft Corporation. Due to the fluctuation of the quality of the Internet, the avatar’s answers to the questions were occasionally delayed. For other procedures (e.g., consent documentation, demographic information input), except the actual interview simulation using avatars, we used supporting materials created using Google form provided by Google Corporation. Participants first completed an informed consent form and then answered questions about demographic information (gender, age, parenting experience, CSA investigation experience). Participants then read short instructions (see Supplementary Appendix 1) on best practice in child forensic interviews and answered two questions to confirm that they had understood these instructions. Participants were asked to read the instructions again if they answered one or both of the two questions incorrectly.

In the present study, six avatars randomly selected from the sixteen avatars were used for each participant. The participants first read the scenario (see Supplementary Appendix 2 for an example) of the alleged case, and answered two questions about their preliminary impression of the case before the interview: (1) the presence of the abuse (“present” or “absent”) and (2) confidence in their assessment on a 6 point scale (“50%: guessing” to “100%: completely sure”). Participants were instructed to focus questions on sexual abuse without further restrictions as to the form of questioning. Each interview lasted up to 10 min. The participants could finish the interview before 10 min has passed, when they thought all information had been elicited from the avatar. The interviews were audio-recorded for coding the questions.

After finishing each interview, participants were again asked to draw a conclusion about the presence of abuse based on the information obtained in the interview and to describe in as much detail as possible what had happened to the avatar. Participants who judged that the avatar had been sexually abused were instructed to describe where, how, and by whom the avatar was abused. Participants who decided that the avatar was not abused were asked to explain what had happened to the avatar that did not constitute abuse. Participants’ conclusions were coded as correct if all information giving rise to the suspicion of sexual abuse was correct, that is, accurate details on who, when, where and what transpired.

In the feedback group, after each of the first to fifth interviews, the participants were given feedback concerning the outcome of the case and concerning the questions (two recommended questions and two not recommended questions) they had asked in the interview. The feedback on questions included presenting selected questions and then explaining how the use of this question type affects the accuracy of answers elicited from a child. Regarding the question types covered by the four pieces of feedback, operators prioritized different types of questions from those that had already been mentioned during the previous feedback occasions.

#### Statistical Analyses

**FIGURE 2:**
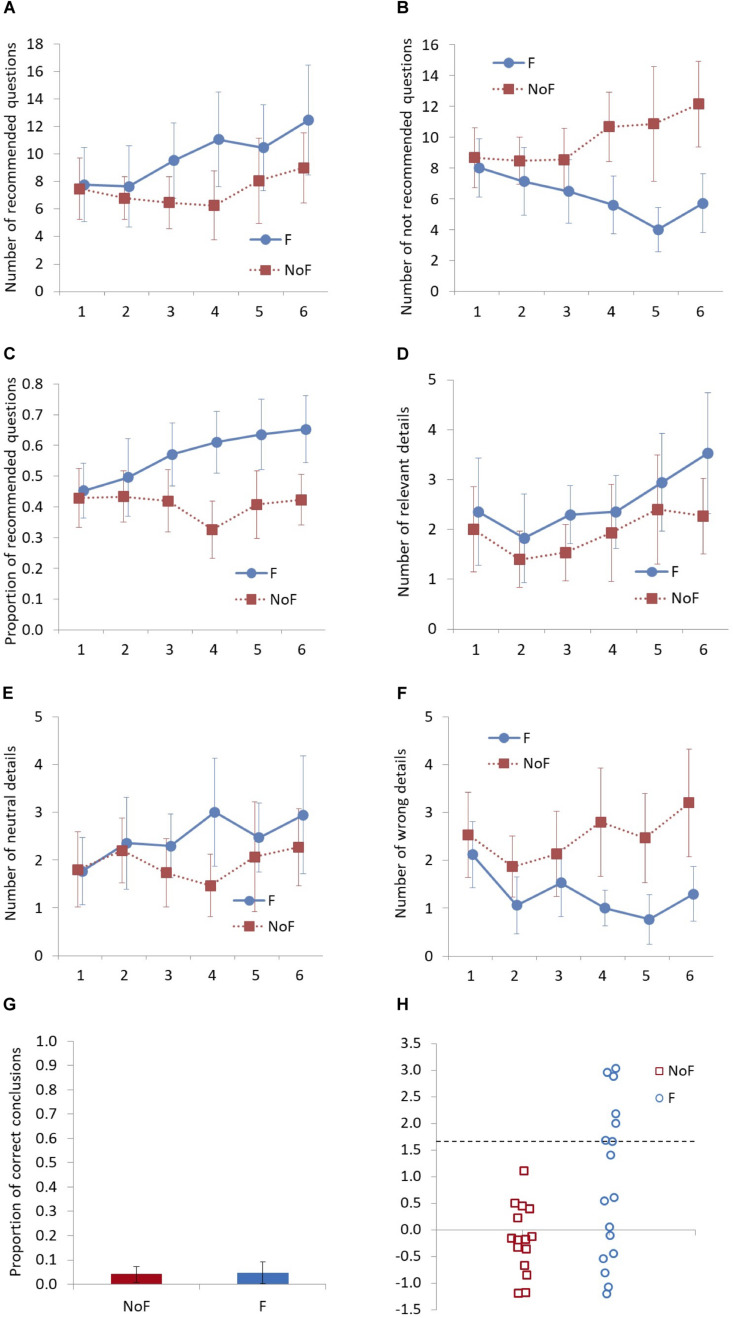
Interview quality, elicited details, the proportion of correct conclusions, and reliable change by group. In **(A–F)**, the *x*-axis displays the number of interviews. In **(G)**, participants’ conclusions were coded as correct when all information giving rise to the suspicion of sexual abuse was correct. In **(H)**, markers above the broken line display reliable change.

Before separate analyses of dependent variables, an integrated factor analysis of six dependent variables (number of recommended questions, number of not recommended questions, proportion of recommended questions, number of relevant details, number of neutral details, and number of incorrect details) was created by calculating the mean of standardized scores within each dependent variable (reversed scores were used for the number of not recommended questions and number of incorrect details). A 2 (feedback: with and without; between-participants) × 6 (number of interviews: first to sixth; within-participants) mixed design two-way analysis of variance (ANOVA) was conducted on the integrated factor to estimate the global effect of training. To check whether the training with feedback improved the interview quality (hypothesis 1) and the number of elicited details compared to training without feedback (hypothesis 2), a series of 2 (feedback: with and without; between-participants) × 6 (number of interviews: first to sixth; within-participants) mixed design two-way ANOVAs were performed on the number of recommended questions (Figure 2A), number of not recommended questions (Figure 2B), proportion of recommended questions (Figure 2C), number of relevant details (Figure 2D), number of neutral details (Figure 2E), and number of incorrect details (Figure 2F). Planned comparisons were used to identify at which interview the difference in interview quality between the two groups became significant (hypothesis 3). Multiple comparisons of the groups at each level of time using Holm’s method (Holm, 1979) were conducted for all the measurements. Mendoza’s multi-sample sphericity test showed that the assumption for ANOVA was violated for all dependent variables except for the number of not recommended questions. The degrees of freedom were corrected using the Huynh-Feldt correction in the analyses of the number of relevant details and neutral details since their epsilon (ε) values were >0.75. Greenhouse–Geisser correction for the degrees of freedom was used for the integrated factor, the number and proportion of recommended questions, and the number of incorrect details since their epsilon (ε) values were <0.75 (Girden, 1992). We used one-way ANOVA to analyze the difference in the proportion of correct conclusions in the second and subsequent interviews between the presence or absence of feedback (hypothesis 4, Figure 2G). In addition, we calculated reliable change indices (RCI) for the percentage of recommended questions to determine how many participants improved their interview quality. The first interview and mean of the last five were compared using the standard deviation of the whole sample for the first interview (i.e., baseline) and the intraclass correlation coefficient of the control group (Chelune et al., 1993), in line with previous Avatar Training studies (Krause et al., 2017; Pompedda et al., 2017). A reliable change was determined using a threshold value of ± 1.645 (90% confidence interval) (Parsons et al., 2009).

A two-sided 95% confidence interval is generally used as the confidence interval of the mean value. However, the overlap between the 95% confidence intervals is roughly equivalent to the test rejecting the null hypothesis H0: μ1 = μ2 and supporting alternative hypothesis H1 μ1 ≠μ2 at the significance level α = 0.005 (Payton et al., 2003). This is a very conservative comparison compared to the commonly used significance level α = 0.05. According to Maghsoodloo and Huang (2010) and Payton et al. (2003), non-overlap of the confidence intervals is comparable to statistical significance at the significance level α = 0.05, by adjusting the confidence intervals according to the ratio of the standard errors of the two samples to be compared. In this method, the larger the ratio of the standard errors, the wider the range of the confidence interval corresponding to α = 0.05 (e.g., *SE* = 1: 83.4%, *SE* = 2: 85.6%, *SE* = 3: 87.9%). In the present study (Figure 2), we employed an 87.9% confidence interval for the number of not recommended questions at the 5th interview (2.6) and the number of incorrect details at the 4th interview (3.0) since their ratios of the standard errors were higher than 2. The ratios of standard errors of the rest of the dependent variables were <2. In these cases, we employed an 85.6% confidence interval corresponding to ratio 2 of standard errors as a slightly more conservative interval.

## Results

### Descriptive Statistics

For the first interview, the overall mean for the number of recommended question was 7.6 (*SD* = 6.4) for the number of recommended questions, 8.3 (*SD* = 5.0) for the number of not recommended questions, 44.1 (*SD* = 23.6) for the proportion of recommended questions, 2.2 (*SD* = 2.5) for the number of relevant details, 1.8 (*SD* = 1.9) for the number of neutral details, and 2.3 (*SD* = 2.0) for the number of incorrect details.

Overall, the participants reached the completely correct conclusion (explanation of what had happened) in 5% (feedback: 5%, Control: 4%) of the cases showing that the task was quite difficult. There was little difference in the proportion of correct dichotomous decisions (present or not present) in the presence of abuse in alleged cases between the feedback group (20%) and the control group (19%).

### Correlations Between Recommended Questions, Details, and Conclusions

Table 2 shows the correlations between the dependent variables. The algorithm worked as expected as the proportion of recommended questions had a significant positive correlation with the number of relevant and neutral details and a significant negative correlation with the number of incorrect details. The reason why the variable correct conclusions was not correlated with the proportion of the recommendation questions may be the low overall proportion (5%) reaching the correct conclusion in the present study.

**TABLE 2:** Correlations between recommended questions, details, and conclusions.

Variables	Relevant details	Neutral details	Incorrect details	Correct conclusion
Number of neutral details	0.65***			
Number of incorrect details	−0.17*	−0.23**		
Correct conclusions	0.12***	0.05**	–0.00	
Recommended questions (%)	0.61***	0.63***	−0.51***	0.02

*p < 0.05, **p < 0.01, ***p < 0.001.

### Integrated Factor

For the integrated factor, no significant main effects were found for feedback [*F*(1, 30) = 3.90, *p* = 0.057, η*_p_*^2^ = 0.12, 1 – β = 0.48] and time [*F*(5, 150) = 1.71, *p* = 0.163, η*_p_*^2^ = 0.05, 1 – β = 0.47] but a significant interaction [*F*(5, 150) = 3.47, *p* = 0.015, η*_p_*^2^ = 0.10, 1 – β = 0.80] was found between feedback and time. The results of planned comparisons revealed larger values in the feedback group than in the control group at the fourth [*F*(1, 30) = 8.05, *p* = 008, *d* = 1.00, 1 – β = 0.87], fifth [*F*(1, 30) = 4.35, *p* = 0.046, *d* = 0.73, 1 – β = 0.65], and sixth [*F*(1, 30) = 5.75, *p* = 0.023, *d* = 0.85, 1 – β = 0.76] interviews, while no significant effects of feedback were found for the first [*F*(1, 30) = 0.15, *p* = 0.706, *d* = 0.14, 1 – β = 0.10], second [*F*(1, 30) = 0.72, *p* = 0.403, *d* = 0.30, 1 – β = 0.21], and third [*F*(1, 30) = 2.55, *p* = 0.121, *d* = 0.57, 1 – β = 0.47] interview.

### Interview Quality

For the number of recommended questions, no significant main effect was found for feedback [*F*(1, 30) = 1.09, *p* = 0.306, η*_p_*^2^ = 0.04, 1 – β = 0.17] but a significant main effect was found for time [*F*(5, 150) = 4.25, *p* = 0.005, η*_p_*^2^ = 0.12, 1 – β = 0.88] while there was no significant interaction [*F*(5, 150) = 1.82, *p* = 0.140, η*_p_*^2^ = 0.06, 1 – β = 0.50] between feedback and time. The results of the planned comparisons also revealed no significant main effects of feedback over time [first: *F*(1, 30) = 0.02, *p* = 0.898, *d* = 0.05, 1 – β = 0.06, second: *F*(1, 30) = 0.14, *p* = 0.711, *d* = 0.14, 1 – β = 0.10, third: *F*(1, 30) = 1.90, *p* = 0.178, *d* = 0.49, 1 – β = 0.39, fourth: *F*(1, 30) = 2.87, *p* = 0.101, *d* = 0.61, 1 – β = 0.51, fifth: *F*(1, 30) = 0.70, *p* = 0.409, *d* = 0.30, 1 – β = 0.20, sixth: *F*(1, 30) = 1.20, *p* = 0.282, *d* = 0.39, 1 – β = 0.29].

For the number of not recommended questions, a significant main effect was found for feedback [*F*(1, 30) = 4.96, *p* = 0.034, η*_p_*^2^ = 0.14, 1 – β = 0.58], no significant main effect was found for time [*F*(5, 150) = 1.00, *p* = 0.419, η*_p_*^2^ = 0.03, 1 – β = 0.35]. There was also a significant interaction between feedback and time [*F*(5, 150) = 5.82, *p* < 0.001, η*_p_*^2^ = 0.16, 1 – β > 0.99]. The results of the planned comparisons revealed a larger number of not recommended questions in the feedback group than in the control group at the fourth [*F*(1, 30) = 7.26, *p* = 0.011, *d* = 0.95, 1 – β = 0.84], fifth [*F*(1, 30) = 8.84, *p* = 0.006, *d* = 1.03, 1 – β = 0.88], and sixth [*F*(1, 30) = 8.98, *p* = 0.005, *d* = 1.05, 1 – β = 0.90] interviews while no significant effects of feedback were found for the first [*F*(1, 30) = 0.14, *p* = 0.708, *d* = 0.13, 1 – β = 0.10], second [*F*(1, 30)= 0.58, *p* = 0.453, *d* = 0.27, 1 – β = 0.19], and third [*F*(1, 30) = 1.21, *p* = 0.280, *d* = 0.39, 1 – β = 0.28] interviews.

For the proportion of recommended questions, no significant main effect of feedback [*F*(5, 150) = 3.82, *p* = 0.060, η*_p_*^2^ = 0.11, 1 – β = 0.47] was found, but a significant main effect for time [*F*(5, 150) = 2.41, *p* = 0.039, η*_p_*^2^ = 0.07, 1 – β = 0.64], and a significant interaction between feedback and time [*F*(5, 150) = 4.66, *p* = 0.002, η*_p_*^2^ = 0.13, 1 – β = 0.97] were found. The results of the planned comparisons revealed a larger proportion of recommended questions in the feedback group than in the control group at the fourth [*F*(1, 30) = 10.12, *p* = 0.003, *d* = 0.1.13, 1 – β = 0.93], fifth [*F*(1, 30) = 4.79, *p* = 0.037, *d* = 0.77, 1 – β = 0.69], and sixth [*F*(1, 30) = 6.42, *p* = 0.017, *d* = 0.91, 1 – β = 0.80] interviews while no significant effects of feedback were found for the first [*F*(1, 30) = 0.08, *p* = 0.778, *d* = 0.10, 1 – β = 0.09], second [*F*(1, 30) = 0.38, *p* = 0.543, *d* = 0.22, 1 – β = 0.15], and third [*F*(1, 30) = 2.61, *p* = 0.117, *d* = 0.58, 1 – β = 0.48] interviews.

### Number of Details Elicited From the Avatars and Proportion of Correct Conclusions

For the relevant details, no significant main effect of feedback [*F*(1, 30) = 1.02, *p* = 0.321, η*_p_*^2^ = 0.03, 1 – β = 0.16] was found, but a significant main effect was found for time [*F*(5, 150) = 2.63, *p* = 0.029, η*_p_*^2^ = 0.08, 1 – β = 0.77]. There was no significant interaction [*F*(5, 150) = 0.34, *p* = 0.875, η*_p_*^2^ = 0.01, 1 – β = 0.13] between feedback and time. The results of the planned comparisons revealed no significant differences between groups over time [first: *F*(1, 30) = 0.15, *p* = 0.701, *d* = 0.14, 1 – β = 0.10, second: *F*(1, 30) = 0.36, *p* = 0.551, *d* = 0.22, 1 – β = 0.15, third: *F*(1, 30) = 2.06, *p* = 0.162, *d* = 0.51, 1 – β = 0.41, fourth: *F*(1, 30) = 0.29, *p* = 0.594, *d* = 0.19, 1 – β = 0.13, fifth: *F*(1, 30) = 0.32, *p* = 0.574, *d* = 0.20, 1 – β = 0.14, sixth: *F*(1, 30) = 1.72, *p* = 0.199, *d* = 0.47, 1 – β = 0.37].

For the neutral details, no significant main effect was found for feedback [*F*(1, 30) = 0.77, *p* = 0.387, η*_p_*^2^ = 0.03, 1 – β = 0.14] or time [*F*(1, 30) = 0.95, *p* = 0.447, η*_p_*^2^ = 0.03, 1 – β = 0.32], and there was also no significant interaction [*F*(5, 150) = 0.93, *p* = 0.459, η*_p_*^2^ = 0.03, 1 – β = 0.32 between feedback and time. The results of the planned comparisons revealed no significant differences between groups over time [first: *F*(1, 30) = 0.01, *p* = 0.959, *d* = 0.02, 1 – β = 0.06, second: *F*(1, 30) = 0.04, *p* = 0.846, *d* = 0.07, 1 – β = 0.07, third: *F*(1, 30) = 0.78, *p* = 0.384, *d* = 0.31, 1 – β = 0.22, fourth: *F*(1, 30) = 3.06, *p* = 0.091, *d* = 0.63, 1 – β = 0.54, fifth: *F*(1, 30) = 0.22, *p* = 0.640, *d* = 0.17, 1 – β = 0.12, sixth: *F*(1, 30) = 0.47, *p* = 0.469, *d* = 0.25, 1 – β = 0.17].

For the incorrect details, a significant main effect was found for feedback [*F*(1, 30) = 6.29, *p* = 0.018, η*_p_*^2^ = 0.17, 1 – β = 0.68], but no significant main effect of time [*F*(5, 150) = 1.51, *p* = 0.206, η*_p_*^2^ = 0.05, 1 – β = 0.44] and no significant interaction [*F*(5, 150) = 1.46, *p* = 0.221, η*_p_*^2^ = 0.05, 1 – β = 0.43] between feedback and time were found. The results of the planned comparisons revealed a smaller number of incorrect details in the feedback group than in the control group at the fourth [*F*(1, 30) = 6.89, *p* = 0.014, *d* = 0.91, 1 – β = 0.80], fifth [*F*(1, 30) = 6.48, *p* = 0.016, *d* = 0.89, 1 – β = 0.79], and sixth [*F*(1, 30) = 5.87, *p* = 0.022, *d* = 0.84, 1 – β = 0.75 interviews while no significant effects of feedback were found for the first [*F*(1, 30) = 0.33, *p* = 0.570, *d* = 0.20, 1 – β = 0.14], second *F*(1, 30) = 2.03, *p* = 0.164, *d* = 0.51, 1 – β = 0.40], and third [*F*(1, 30) = 0.68, *p* = 0.416, *d* = 0.29, 1 – β = 0.20] interviews.

For the proportion of correct conclusions (see Figure 2G), no significant main effect [*F*(1, 30) = 0.02, *p* = 0.896, *d* = 0.07, 1 – β = 0.07] was found.

### Reliable Change Analysis

None of the participants in the control group had reliable changes in the proportion of recommended questions, as expected. Of the participants in the control group, 67% (*n* = 10) decreased the proportion of recommended questions. In contrast, 65% (*n* = 11) in the feedback group increased the proportion of recommended questions, and 41% (*n* = 7) in the feedback group had reliably changed their proportion of recommended questions in the second and subsequent interviews from the first interview (see Figure 2H).

## Discussion

### Differences With Previous Results and Possible Cultural Effects

In the present study, an integrated factor combining information from all dependent variables demonstrated an interaction between group and time, and results of planned comparisons demonstrated better interview quality in the group receiving (vs. not receiving) feedback from the fourth interview onward, while there was no difference between groups at the baseline. This indicates that Avatar Training may require at least four simulated interviews to obtain a significant effect on trainees.

The proportions of recommended questions were significantly different between the groups. From the results of planned comparisons of the proportion of recommended questions, it can be seen that the difference in interview quality between the groups receiving and not receiving feedback appeared from the fourth interview, as observed in the integrated factor. This is in line with findings in previous studies (Pompedda et al., 2015, 2017; Krause et al., 2017) that tested Avatar Training. In the present study, more participants in the feedback group achieved a reliable change (41%) compared to those in previous studies (Krause et al., 2017: 32%, Pompedda et al., 2015: 33%). This means that Avatar Training can be administered online and in a non-Western context achieving impressive changes in interviewer behavior.

Although the results of the present study proved generalizability across the administration method and cultural setting of the Avatar Training, we also found some differences in results between the present study and previous Avatar Training studies. In terms of interview quality, Pompedda et al. (2015) found significant differences between groups in the number of recommended and not recommended questions, but the present study showed significant differences only in the number of not recommended questions. The improvement of interview quality was, therefore, derived from the change in the number of not recommended questions in the present study. This tendency is likely linked to the results regarding elicited details as well. A significant difference between groups was found in the number of incorrect details, but no significant difference was found in the number of relevant and neutral details. In addition, the low number of relevant and neutral details resulted in a low proportion of correct conclusions. Previous studies (Pompedda et al., 2015; Krause et al., 2017) demonstrated improvements in eliciting both relevant and neutral details and in the proportion of correct conclusions; the finding that the improvement of interview quality pertained only to the aspect of decreasing not recommended questions is unique to the present study.

However, we should highlight the possibility that a superficial difference between the present and previous studies might emerge due to the present study’s relatively low statistical power. As described, only 11 out of 64 analyses had 0.80 or higher power. This indicates that the sample size in the present study was on the low side for many tests, and non-significant results in elicited relevant and neutral details might be due to this limitation. However, with regards to the comparison of effect sizes, the main effects of factors (group and time) and their interaction in ANOVA on relevant (η*_p_*^2^ = 0.01–0.08) and neutral details (η*_p_*^2^ = 0.03) were smaller than those of Krause et al. (2017) (relevant details: η*_p_*^2^ = 0.13–0.24; neutral details: η*_p_*^2^ = 0.12–0.21) and Pompedda et al. (2017) (relevant details: *d* = 0.27; neutral details: *d* = 0.43). Moreover, it is important to note that the present study did not show an increase in correct conclusions in the feedback group relative to the control group, while previous studies did. Considering these comparisons to previous research, possible explanations for unique results of the present study are warranted.

Under the assumption of differences in the number of recommended questions between the present and previous studies, a factor possibly influencing the results is culture. The cross-cultural review of self-view (Heine et al., 1999) suggested the possibility that Japanese interviewers might be more likely to accept negative feedback while they might discount positive feedback (Heine et al., 1999). Thus, the feedback group in the present study might place a higher value on negative feedback in the Avatar Training, and that tendency might influence the results of recommended and not recommended questions.

Additionally, there was a difference in the total number of questions asked between the present study and previous studies. The participants in this research asked quite a low number of questions (*M* = 16.6, *SD* = 8.5) compared to those in previous research (around 20 to 40 questions, see Figure 1 in Pompedda et al., 2015). One possible explanation for this difference may be cultural differences in the significance attached to making mistakes. Psychological findings (Lutwak et al., 1998; Bear et al., 2009; Furukawa et al., 2012) have indicated that populations with a background of Asian culture are more prone to shame in contrast to other populations. Face-saving plays an important role in emotional situations in the Japanese cultural context, and Japanese people feel shame as a result of losing face in public (Boiger et al., 2013). In the present study, participants more prone to shame might ask fewer questions to avoid errors (i.e., to avoid asking not recommended questions). In addition, this tendency may have been enhanced by the fact that 72% (*n* = 23) of the participants in the present study were women, as women tend to experience shame more than men (Kitayama et al., 1997; Hasui et al., 2009).

A lack of improvement in the number of recommended questions may be related to the mechanism of delivering feedback. In studies comparing face-to-face and audio-only interactions, no difference in the effects of psychotherapy (Day and Schneider, 2002) and cooperative learning (Roseth et al., 2011) was found, and the communication actually increased in audio-only compared to face-to-face situations (Doherty-Sneddon et al., 1997). However, visual signals such as eye gaze and head nodding have been suggested to play an important role in interaction (Duncan, 1972; Clark and Schaefer, 1989; Clark and Brennan, 1991), and explicit verbal elicitation of feedback is needed more frequently when visual signals are unavailable (Doherty-Sneddon et al., 1997). In the present study, feedback conversations were semi-structured (explanation of two recommended questions and two not recommended questions), and the number of utterances was controlled so as not to differ significantly from the previous Avatar Training research. Therefore, limiting the amount of communication under the remote training condition using audio (i.e., the condition where visual signals were not available) to the same level as face-to-face might have diminished the training results obtained.

### Limitations and Future Implications

As previously described, a limitation of the present study that must be initially highlighted is the relatively small size of the sample. This resulted in lower statistical power, making it more challenging to interpret non-significant findings. Further studies may therefore be required to determine the effectiveness of Avatar Training in Japan and remote administration.

Nevertheless, the present study’s results expand knowledge about the efficacy of simulated training, and illustrate implications for future research. While the interview quality was improved in the present study, we should note that the Avatar Training did not improve the participants’ capacity to reach correct conclusions. Repeated failure in reaching correct conclusions may have affected the motivation of the participants negatively. These problems may be counteracted by adding another intervention. For instance, Krause et al. (2017) tested the effectiveness of the combination of feedback and reflection, but it was not clearly better than training with feedback alone. In another context, learners’ preference for one of two different teaching methods (analytic vs. global: Abraham, 1985) in the context of grammar teaching might be considered to point to an intervention that improves training results in Japanese interviewers. The analytic style is defined as rule-oriented and deductive learning, while the global style is defined as non-rule oriented and inductive learning. Feedback used in the present study can be categorized as a global learning style because it is the inductive process of learning the types of recommended and not recommended questions from the feedback on the questions used in the simulated interviews. Since there may be a relationship between the preference for learning styles and cultural background (Oxford and Anderson, 1995), it can be assumed that the effectiveness of a learning style varies between different cultural settings. Therefore, adding rule-oriented components might be a shortcut to make the intervention more effective for Japanese trainees.

In an investigation of the training effect, a follow-up exploration should also be conducted to assess transfer of the training effect. Moreover, considering the low cost of conducting Avatar Training, retention of learned skills using booster sessions after an interval from the first training is also a relevant topic to be tested in future research. If booster sessions are found to be sufficient to maintain interview skills, Avatar Training can be expected to be established as a training protocol that continuously contributes to interviewer training.

We also need to consider expanding the coverage of interview skills included in the Avatar Training. Recently, the revised protocol of NICHD emphasized supportive interviewing (Hershkowitz et al., 2017), and field interviews with physically abused children in Israel proved that supportive statements decreased children’s reluctance and resulted in increased informativeness (Blasbalg et al., 2018). Future studies should address more comprehensive interview skills, including both question types and support.

Last but not least, the length (2–2.5 h) of the current Avatar Training may be too long to be implemented in all professional settings. A shortened interview (e.g., a 5 min interview instead of a 10 min interview) may be tested to expand the generalizability of the Avatar Training. In that case, a limited number of questions can be asked compared to 10 min of interviews, which may decrease the probability of obtaining relevant and neutral details. To improve the possibility of reaching correct conclusions in the short interview, this issue may need to be examined using avatars that are less reluctant to disclose abuse.

## Data Availability Statement

The datasets generated for this study will not be made publicly available since the agreements of the participants has not been provided on sharing the datasets to the persons who are not included in the project. Requests to access the datasets should be directed to the corresponding author.

## Ethics Statement

The studies involving human participants were reviewed and approved by Psychology Major Ethics Committee at Hosei University. Written informed consent for participation was not required for this study in accordance with the national legislation and the institutional requirements.

## Author Contributions

SH, SY, FP, JA, and PS contributed to the conception and design of the study. SH and SY collected the data. PS and MN supervised data collection. SH performed the statistical analyses and wrote the first draft. All authors contributed to the interpretation of the analyses, contributed to the revision of the manuscript, edited and gave final approval for publication and were accountable for this work.

## Conflict of Interest

The authors declare that the research was conducted in the absence of any commercial or financial relationships that could be construed as a potential conflict of interest.
