# Supplementary material for: Online Simulation Training of Child Sexual Abuse Interviews With Feedback Improves Interview Quality in Japanese University Students
Source: Front Psychol. 2020 May 26;11:998. doi: 10.3389/fpsyg.2020.00998 (PMC7265454; doi:10.3389/fpsyg.2020.00998)
Supplement: Supplementary file 1 [file Data_Sheet_1.docx]

# Appendix 1. *Instruction about best interview practice*

**Guidelines for the correct questioning style**

The behavior of children in interviews is considerably different from that of adults. Young children are especially vulnerable to suggestion: They tend to perceive any information provided by adults as true and do not always negate incorrect statements made by the interviewer. Because of that, there are established recommendations about question types to use when interviewing children: In general, open-ended question types are recommended. This is because they are most likely to elicit a reliable answer. Open-ended questions can be:

*Invitations*: Open-ended questions (questions, statements or imperatives) used to elicit free recall responses from the child. Invitations could be general (‘Tell me everything that happened from the very beginning to the end’) or relate to something just mentioned by the child (‘Tell me more about that’).

*Facilitators*: Non-suggestive encouragements to continue with a response. These include utterances like ‘ok,’ and restatements (echoing) of the child’s previous utterance.

*Directive questions*: These refocus the child’s attention on details already mentioned by the child and request further elaboration (for example, ‘Where were you when that happened?’).

On the other hand, closed and suggestive questions should be avoided, as the answers elicited by them are much less reliable and they might create false memories in children. Closed and suggestive questions include:

*Option-posing questions*. These focus the child’s attention on issues that the child had not previously mentioned but do not imply that a particular response is expected. The answer to these types of questions is usually ‘yes or no’. For example, the interviewer might ask ‘Did he touch your penis?’ or ‘Did he do anything with his penis?’

*Suggestive questions*. These are stated in such a way that the interviewer strongly communicates what response was expected (for example: ‘He forced you to do that, didn’t he?’), or assumed details that had not been revealed by the child (for example: Child: ‘We laid on the sofa.’ Interviewer: ‘He laid on you or you laid on him?’).

**Questions before the interview:**

1. If the child doesn’t provide any detail regarding the alleged circumstances of abuse, the interviewer should ask the child questions related to the alleged situation. For example: Did your father touch you?

◯ Yes

◯ No

2. If the child provides a detail regarding the alleged events, for example ‘he punched me’ which is the best question to ask?

◯ Did it hurt?

◯ Who punched you?

◯ Was it your father?

# Appendix 2. *Background to the interview*

**Haruna (4 years old)**

Haruna is a 4-year-old girl. She is outgoing, although she has some developmental cognitive delays. She lives with her father, mother and brother Yota (12 years old). Occasionally her aunt also stays with them. She doesn't have an ideal family environment, due to the low educational level of her parents, and the fact that her mother (Narumi) is mentally disabled. Haruna does not go to kindergarten, since her father doesn't allow her to. Haruna's father (Tatsuya) is not her biological father, but he married Haruna’s mother after Haruna's birth.

Yota takes care of their mother, along with the aunt, while his father takes care of Haruna. Yota and the aunt noticed some bruises on Haruna’s arms. They went to a doctor asking for a cure for those bruises, and the doctor did not exclude the possibility of abuse.
